# Improved parameter-estimation with combined PET-MRI kinetic modelling

**DOI:** 10.1186/2197-7364-2-S1-A25

**Published:** 2015-05-18

**Authors:** Kjell Erlandsson, Maria Liljeroth, David Atkinson, Simon Arridge, Sebastien Ourselin, Brian Hutton

**Affiliations:** Institute of Nuclear Medicine, University College London, London, UK; Centre for Medical Imaging, University College London, London, UK; Department of Computer Science, University College London, London, UK; Centre for Medical Imaging Computing, University College London, London, UK; The Centre for Medical Radiation Physics at the University of Wollongong, Northfields Ave, Wollongong, Australia

Kinetic analysis can be applied both to dynamic PET and dynamic contrast enhanced (DCE) MRI data. We have investigated the potential of combined PET-MRI kinetic modelling using simulated FDG data. The volume of distribution, Ve, for the extra-vascular extra-cellular space (EES) can be estimated by DCE-MRI, and used to reduce the number of parameters in the PET model. We use a 3 tissue-compartment model with 5 rate constants (3TC/5k), in order to distinguish between EES and the intra-cellular space (EIS). In the standard models, k3 represents transfer from the un-metabolised to the metabolised (M) extra- vascular compartment. In our new model, k3’ represents transfer from EES to the EIS M-compartment. We also define the more biologically relevant constant, k3"=Vek3’, to be used together with the true EES tracer-concentration. Time-activity curves were generated using the 3TC/5k-model with 3 different Ve- values, but constant k3". Noise was added and the data were fitted with the 2TC/3k model and with the constrained and-un-constrained 3TC/5k model. 100 noise- realisations were generated at 4 different noise-levels. For the standard 2TC/3k-model, the estimated k3-values were in the range [0.053, 0.094] with SD in the range [0.002,0.043] /min. For the un-constrained 3TC/5k model, the k3"-values were in the range [0.041,0.187] and SD in [0.053,0.208] /min. With fixed Ve the range of k3" is reduced to [0.083,0.091] with SD in [0.002,0.017] /min. The true k3" value was 0.091/min. By incorporating information from DCE-MRI into the PET kinetic model, we obtained a good estimate of the parameter k3", independent of Ve.

